# A Rare Case of Luc’s Abscess in an Immunocompromised Female Patient

**DOI:** 10.7759/cureus.71185

**Published:** 2024-10-10

**Authors:** Sharifah Fatimah Syahirah Syed Afandi, Rohaida Ibrahim, Zalilah Musa, Saadah Mohamad, Salina Husain

**Affiliations:** 1 Department of Otorhinolaryngology-Head and Neck Surgery, Faculty of Medicine, Universiti Kebangsaan Malaysia, Kuala Lumpur, MYS; 2 Department of Otorhinolaryngology, Hospital Sultanah Nur Zahirah, Kuala Terengganu, MYS

**Keywords:** complication, luc’s abscess, mastoiditis, otitis media, subperiosteal

## Abstract

Although Luc’s abscess represents a rare complication of otitis media, it is an important complication to be considered by clinicians, by which infection spreads from the middle ear and can result in subperiosteal collection beneath the temporal muscle. Because of its rare occurrence, the diagnosis and treatment might be delayed. Here, we report the case of a 48-year-old female with Luc’s abscess with the involvement of the mastoid bone and discuss its clinical presentation and successful management.

## Introduction

In the early 20th century, Henry Luc, a French clinician, described a subperiosteal abscess located deep in the temporalis muscle as a complication of acute otitis media in a nine-year-old girl [1-3]. Luc’s abscess is rarely encountered in the post-antibiotic era [2]. Given its rarity, clinicians, especially primary care doctors and general practitioners, may not be aware of this complication. This can result in delayed diagnosis and management, subsequently causing serious harm to the patient.

Luc’s abscess differs from other subperiosteal abscesses due to the spread of a middle ear infection through a pre-existing pathway in the external ear canal [4]. Unlike other subperiosteal abscesses of otitic origin, it usually develops after the spread of the infection to the subperiosteal area from cortical bone destruction, often as a result of acute mastoiditis [4]. Subperiosteal abscesses with acute mastoiditis can occur through a rare route of infection where the spread to the temporal fossa occurs via the pneumatized zygomatic arch [4]. Computed tomography (CT) scan is important to see the extension of the disease. Here, we report a case of acute otitis media complicated by Luc’s abscess with the involvement of the mastoid bone.

## Case presentation

**Figure 1:**
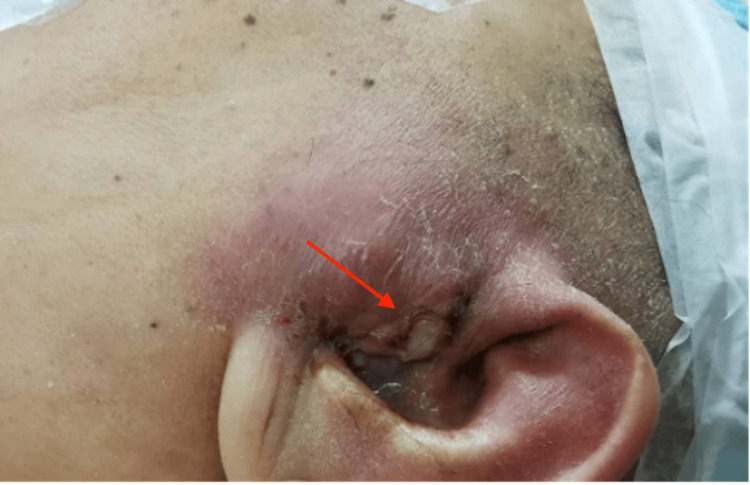
The left temporal swelling extended anteriorly to the left zygoma with punctum over the left tragus (red arrow).

A 48-year-old female with underlying uncontrolled diabetes mellitus, end-stage renal failure, and hypertension presented with complaints of left otalgia associated with purulent left ear discharge for two weeks. She also complained of reduced hearing in the same ear. Subsequently, she developed left temporal swelling for two days before admission. Physical examination showed erythematous, firm, warm, and tender swelling on the left temporozygomatic region. There was pus discharge draining from the ruptured punctum at the tragus of the left ear with slough tissue underneath (Figure 1). An otoscopic examination of the left ear showed an edematous external auditory canal with pus discharge. Otherwise, the facial nerve was intact, the mastoid area was normal, and the post-auricular sulcus was not obliterated.

Blood investigations revealed leukocytosis with neutrophil predominance, hypochromic normocytic anemia, thrombocytosis, elevated C-reactive protein, and elevated erythrocyte sedimentation rate (Table 1). The estimated glomerular filtration rate was 10 mL/minute/1.73m^2^.

**Table 1:** Blood parameters of our patient.

Blood parameter	Value	Normal range
Hemoglobin	74.0 g/L	120.0–150.0 g/L
Mean corpuscular volume	80.4 fL	84–94 fL
Mean corpuscular hemoglobin concentration	291 g/L	315.0–345.0 g/L
White blood cells	20.5 × 10^9^/L	4.0–10.0 × 10^9^/L
Neutrophils	86%	60–80%
Platelet	471 × 10^9^/L	150–410 × 10^9^/L
C-reactive protein	105.8 mg/L	<5 mg/L
Erythrocyte sedimentation rate	120 mm/hour	<20 mm/hour

**Figure 2:**
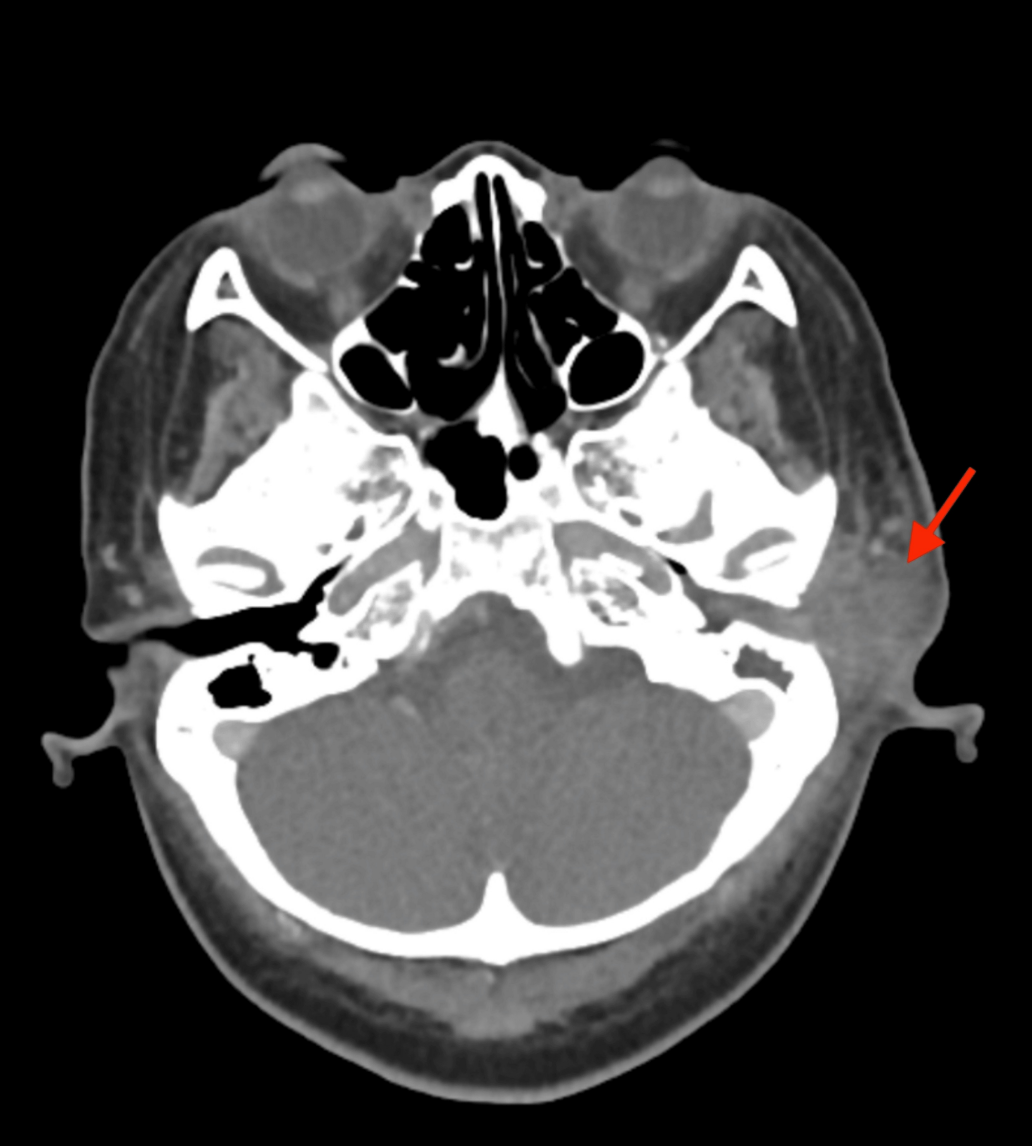
Ill-defined mildly enhancing soft tissue lesion seen extending from the left pre-auricular region into the external auditory canal (red arrow).

**Figure 3:**
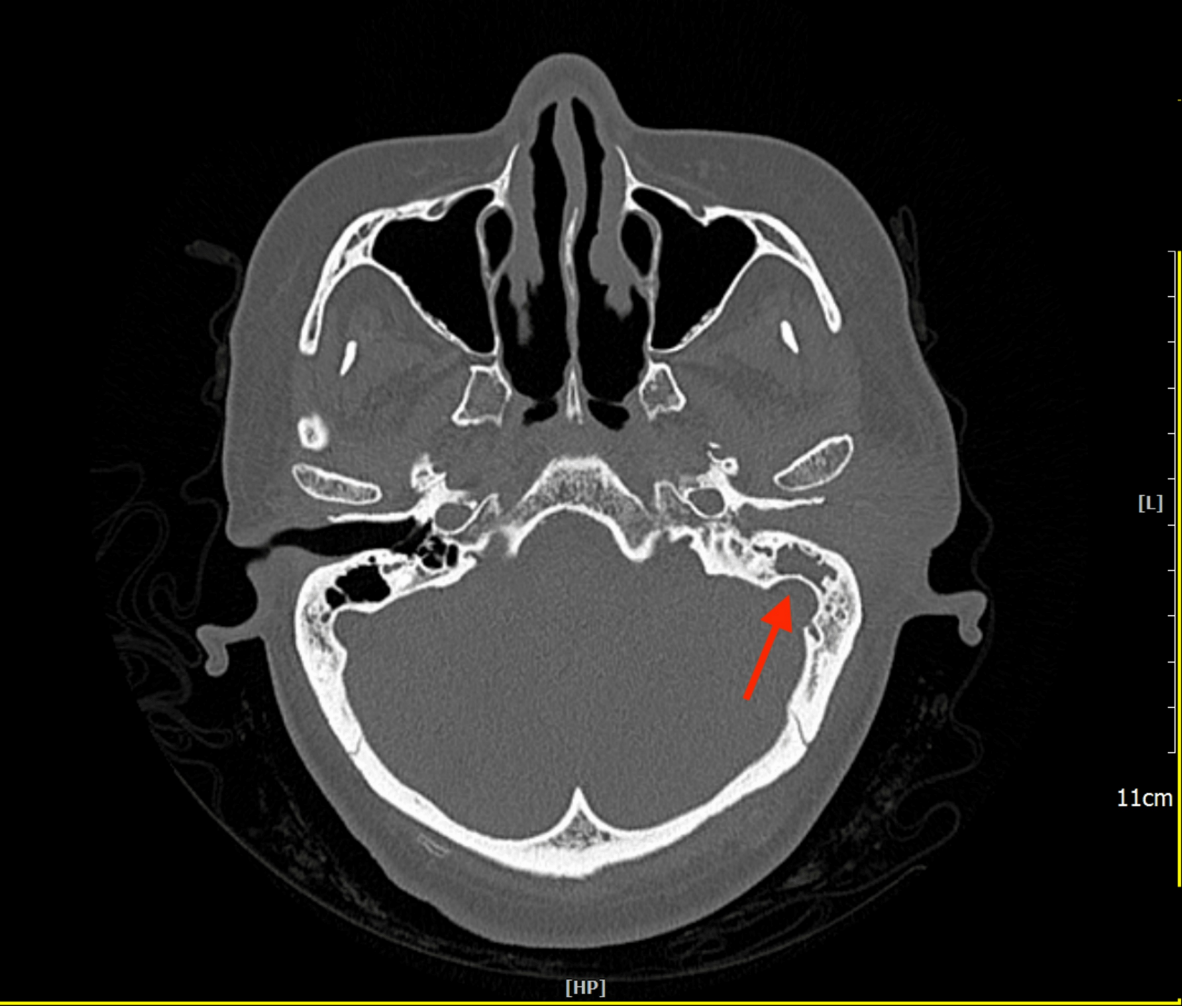
The mastoid septa are eroded and the mastoid bone is mildly sclerotic (red arrow).

The baseline pure tone audiometry test showed a mild level of conductive hearing loss on the left side. A High-resolution computed tomography (HRCT) scan of the temporal bone demonstrated an ill-defined mildly enhancing soft tissue lesion seen extending from the left pre-auricular region into the external auditory canal (Figures 2, 3). It measured approximately 2.8 × 4.2 × 2.8 cm (anteroposterior × transverse × craniocaudal). Hypodense soft density is also seen occupying the middle ear canal and mastoid air spaces. The tympanic membrane was not visualized. The ossicles were intact. The mastoid septa were eroded. The mastoid bone was mildly sclerotic. No tegmen tympani erosion was noted. Cochlear and semicircular canals were normal in configuration. The bony facial nerve canal was intact. A working diagnosis of left Luc’s abscess with left mastoiditis was made.

Incision and drainage of the abscess and cortical mastoidectomy were performed. Intraoperatively, 2 cc of pus was drained out. Mastoid cells were sclerotic and granulation tissue filled in the mastoid air cells until the middle ear and superiorly to the incus.

No cholesteatoma sac was seen. Culture from the abscess and tissue revealed *Pseudomonas aeruginosa* sensitive to imipenem. The patient was treated with a 14-day course of intravenous imipenem 250 mg three times a day (renal-adjusted dose). Postoperative recovery was uneventful and she was discharged after completing a 14-day course of intravenous antibiotics. Follow-up at the otorhinolaryngology clinic showed regression of Luc’s abscess.

## Discussion

In the literature, the number of Luc’s abscess cases that have been reported is very limited. Literature review shows that numerous cases of Luc’s abscess have been reported in the male and pediatric age groups [4,5]. We reported a case of Luc’s abscess in an adult, female, and immunocompromised patient. The most common symptoms of Luc’s abscess include ear pain and swelling around the zygomatic region, accompanied by fever and malaise [6,7]. Swelling associated with Luc’s abscess can occur in different locations, such as the preauricular area, temporal region, cheek, eyelids, and mastoid area, with the frequency decreasing in that order [6,7]. Our patient presented with otalgia and temporozygomatic swelling.

Diagnosing Luc’s abscess could be challenging as it is rarely encountered by clinicians in the post-antibiotic era. Given its rare occurrence, the diagnosis and treatment might be delayed which can lead to serious harm to the patient. In our case, the patient had underlying comorbidities, including diabetes mellitus and end-stage renal failure which contributed to a higher frequency of infections and complications.

Complications of middle ear infections are classified into intracranial and extracranial complications [3]. Intracranial complications can be subdivided into intracerebral and extracerebral complications, while extracranial complications are further subdivided into intratemporal and extratemporal complications. Intracerebral complications of otitis media are focal encephalitis, cerebral abscess, and otitic hydrocephalus while extracerebral complications are epidural abscess, subdural abscess, lateral sinus thrombosis, and meningitis. Intratemporal complications of otitis media include mastoiditis, petrositis, labyrinthitis, labyrinthine fistula, facial nerve palsy, and ossicular necrosis while extratemporal complications are Bezold’s abscess, Citelli’s abscess, post-auricular abscess, zygomatic abscess, and Luc’s abscess. Extracranial, extratemporal spread of disease comprises subperiosteal abscess formation in different areas of the temporal bone [3]. Generally, intraosseous suppuration causes osteitis, most commonly in the mastoid, that leads to pus collection and causes destruction of the cortex overlying the affected air cells [3]. Pus then spreads between cortical bone and the periosteum, leading to the formation of a subperiosteal abscess [3]. The periosteum, at least temporarily, acts as a solid barrier against further progression of infection [3]. An abscess that is formed in this manner is a complication of mastoiditis, which itself is a complication of otitis media [3].

The most common suppurative complication of acute otitis media is acute mastoiditis [8]. Acute mastoiditis can later progress to a subperiosteal abscess which usually requires surgical drainage by some form of a mastoidectomy. In 1913, Henry Luc first described that subperiosteal abscess is related to acute otitis media [1]. Luc’s abscess can develop without the involvement of the mastoid bone. The infection spreads through the notch of Rivinus and the branches of the deep auricular arteries, which are situated between the roof of the middle ear and the external ear canal and the subperiosteal area [8]. According to Luc, if mastoiditis is absent, patients do not require mastoidectomy, a procedure known for its associated risks [4]. However, The validity of Luc’s theory has been challenged due to a growing number of cases where both children and adults present with a subperiosteal abscess beneath the temporalis muscle alongside mastoiditis [9]. This has been proven through case reports gathered from 1989 to 2018, involving a total of 21 patients (17 children and 4 adults) [9]. The findings indicated that nearly all patients (95.2%) except one exhibited signs of mastoiditis on a CT scan [9], thereby contradicting Luc’s theory.

This report describes an immunocompromised patient presenting with acute otitis media, complicated by Luc’s abscess, in the presence of otomastoiditis. She presented with moderate signs of infection and inflammation (local fluctuation with pus discharge, leukocytosis, thrombocytosis, elevated erythrocyte sedimentation rate, and C-reactive protein). The management of this patient included medical therapy (systemic and local antibiotic), surgical drainage, and cortical mastoidectomy, guided by the findings on the CT scan.

Luc’s abscess is associated with relatively little morbidity and requires more limited surgical intervention [9]. HRCT temporal scan was the imaging of choice for this patient. This imaging is crucial for assessing the extent of the disease, regardless of mastoid involvement, as well as for determining the appropriate surgical approach for this patient [8].

For cases of coalescent mastoiditis or cortical erosion of the mastoid, mastoidectomy is recommended, particularly in children if there is no improvement after 48 hours of intravenous empirical antibiotics [9]. The most common organisms causing Luc’s abscess include *Streptococcus* species, *Staphylococcus aureus*, and anaerobic bacteria. *Pseudomonas aeruginosa* can also be a causative organism of Luc’s abscess but it is less common and typically associated with immunocompromised patients. In our case, an HRCT temporal scan showed erosion of the left mastoid septa and sclerotic mastoid bone with soft tissue density within the middle ear and mastoid air spaces, possibly due to the virulence of the bacterium isolated. The surgical approach was the treatment of choice for this patient. Local incision and drainage combined with cortical mastoidectomy were performed for this patient as the HRCT temporal scan was suggestive of otomastoiditis.

## Conclusions

Luc’s abscess is an uncommon complication that can occur with otitis media. In the post-antibiotic era, Luc’s abscess has been uncommon and clinicians, particularly primary care doctors and general practitioners, may be unaware of this complication. Unfamiliarity with this treatable complication might lead to delays in making a diagnosis and initiating proper treatment, which can cause severe harm to patients. Immunocompromised patients, such as drug abusers and diabetic patients, are at a higher risk of developing this complication. Imaging with a CT scan is important to evaluate the extent of the abscess and to help in surgical management. A surgical approach together with intravenous anti-biotherapy is the best choice of treatment for a patient with Luc’s abscess with otomastoiditis.
